# Advancements in interventional gastroenterology GI tract cancers: A narrative review

**DOI:** 10.1097/MD.0000000000048554

**Published:** 2026-04-24

**Authors:** Gaurang Hasmukhbhai Suhagiya, F.N.U. Priyanka, Zainab Obaid Ullah, Syed Ahmad Farooqi, Ali Karim, Mubeena Javed, Alfa Almani, Sajid Ali, Allah Warayo, Hafsah Ali, F.N.U. Karishma, Falah Asad, Kishore Kumar, Barkha Goswami, Sana Memon, Mahesh Kumar, Abida Perveen

**Affiliations:** a Department of Medicine, Ibn e Seena Hospital, Kabul, Afghanistan.

**Keywords:** artificial intelligence, EMR, endoscopic ultrasound, ESD, gastrointestinal cancers, interventional gastroenterology

## Abstract

Gastrointestinal (GI) tract cancers remain a leading cause of cancer-related morbidity and mortality worldwide, frequently presenting at advanced stages that limit curative treatment options. Traditional management has relied on surgery and systemic therapies; however, advances in interventional gastroenterology have significantly transformed diagnostic and therapeutic strategies. This narrative review aims to summarize recent advancements in interventional gastroenterology for GI cancers, focusing on evolving diagnostic tools, minimally invasive therapeutic techniques, and emerging technologies shaping modern oncologic care. A narrative review of current literature was conducted to evaluate developments in high-resolution imaging, image-enhanced endoscopy, endoscopic ultrasound, minimally invasive resection methods, ablative therapies, and palliative endoscopic interventions across esophageal, gastric, colorectal, pancreatic, and biliary malignancies. Advanced endoscopic techniques have improved early cancer detection, enabled precise staging, and facilitated organ-preserving treatment for early-stage malignancies. Interventional approaches also provide effective palliation in advanced disease. Emerging innovations, including artificial intelligence, robotic-assisted endoscopy, and endoscopic molecular diagnostics, are expanding the role of endoscopy in personalized cancer management. The integration of advanced interventional gastroenterology into multidisciplinary cancer care represents a paradigm shift toward personalized, minimally invasive management of GI malignancies, with significant potential to improve clinical outcomes and quality of life.

## 1. Introduction

Gastrointestinal (GI) tract cancers – including esophageal, gastric, colorectal, pancreatic, and biliary malignancies – constitute a substantial global health burden and are among the leading causes of cancer-related mortality worldwide.^[1]^ Poor prognosis is frequently attributed to late diagnosis, complex anatomy, and limited access to curative therapies at advanced stages.^[2]^ Historically, management relied primarily on surgical resection and systemic chemotherapy; however, these approaches are often associated with significant morbidity and are unsuitable for many patients.^[3]^

Over the past decade, the rapid evolution of interventional gastroenterology has transformed both diagnostic and therapeutic strategies in GI oncology.^[4]^ Advances in endoscopic imaging, tissue acquisition, minimally invasive resection, and therapeutic interventions have enabled earlier detection, accurate staging, and organ-preserving treatment options with reduced procedural risk.^[5]^ These developments have expanded the role of the gastroenterologist from diagnostician to interventional oncologist.

Innovations such as endoscopic mucosal resection (EMR), endoscopic submucosal dissection (ESD), endoscopic ultrasound (EUS)-guided diagnostic and therapeutic procedures, ablative technologies, and endoscopic stenting now offer curative potential in early-stage disease and effective palliation in advanced cancer.^[6,7]^ This narrative review highlights recent advancements in interventional gastroenterology for GI tract cancers, focusing on evolving technologies, clinical applications, and their impact on contemporary cancer care pathways.

## 2. Methods

This article was conducted as a narrative review based on an expert-driven, nonsystematic assessment of the literature. Relevant studies were identified through searches of PubMed, Scopus, and Web of Science, focusing on English-language publications related to interventional gastroenterology and GI tract cancers.

Priority was given to high-impact reviews, clinical trials, consensus guidelines, and landmark studies emphasizing diagnostic innovation, therapeutic endoscopy, and clinical outcomes. The aim was to synthesize clinically meaningful advancements rather than perform a quantitative or bibliometric analysis. This review does not follow systematic review methodology and was not intended to provide meta-analytic estimates.

## 3. Discussion

### 3.1. Minimally invasive resection techniques

The introduction of endoscopic resection techniques has revolutionized the management of early GI cancers.^[8]^ EMR is widely used for small, superficial neoplastic lesions, particularly in the esophagus, stomach, and colon.^[9]^ In contrast, ESD enables en bloc resection of larger or fibrotic lesions, allowing precise histopathological assessment and lower recurrence rates.^[10]^

Multiple studies have demonstrated that, in carefully selected patients, EMR and ESD achieve oncologic outcomes comparable with surgical resection while preserving organ integrity, reducing complications, and shortening hospital stay.^[11–13]^ These techniques have become standard of care for early-stage mucosal and superficial submucosal cancers in high-volume centers (Table 1).

**Table 1:** Key interventional techniques in GI tract cancers.

Technique	Application	Advantages	Limitations
Endoscopic ultrasound	Tumor staging, tissue diagnosis	Operator-dependent	Operator-dependent
Narrow band imaging	Early neoplasia detection	Enhanced mucosa visualization	Limited penetration depth
Confocal laser endomicroscopy	In vivo histology	Real-time diagnosis	Expansive limited availability
Chromoendoscopy	Lesion characterization	Cost-effective, improves detection	Requires expertise
Autofluorescence imaging	Dysplasia detection	Noninvasive enhanced screening	High false-positive rate

GI = gastrointestinal.

### 3.2. EUS: diagnostic and therapeutic applications

EUS is a cornerstone in the staging and diagnosis of GI malignancies, particularly pancreatic, esophageal, gastric, and rectal cancers.^[14]^ By providing high-resolution imaging of the GI wall layers and surrounding structures, EUS allows accurate T and N staging.^[15]^

EUS-guided fine-needle aspiration and fine-needle biopsy enable tissue acquisition with high diagnostic accuracy and low complication rates.^[16]^ Beyond diagnosis, therapeutic EUS has expanded rapidly, including celiac plexus neurolysis for pancreatic cancer pain, drainage of pancreatic fluid collections, and management of malignant biliary obstruction.^[17,18]^ EUS-guided gastroenterostomy has emerged as a minimally invasive alternative to surgical bypass in malignant gastric outlet obstruction.^[19]^ (Table 2).

**Table 2:** Role of EUS in GI cancer management.

Technique indication	Indication	Benefit	Clinical setting
Endoscopic mucosa resection	Early esophageal, gastric, and colorectal cancers	Minimally invasive, curative in early stages	Outpatient early-stage disease
Endoscopic submucosal dissection	Large or deeper early tumors	En bloc resection lower recurrence	Requires advanced expertise
EUS-guided drainage (biliary pancreatic)	Obstructive jaundice collection	Alternative to surgery or percutaneous drainage	Advanced metastatic disease
Stent placement	Malignant obstruction	Symptom relief, improves quality of life	Palliative care
Radiofrequency ablation	Barrett esophagus with dysplasia	Ablates dysplastic tissues, reduces cancer progression	Surveillance and treatment

EUS = endoscopic ultrasound, GI = gastrointestinal.

### 3.3. Ablative therapies for early GI cancers

Ablative endoscopic therapies provide effective treatment options for early neoplasia and premalignant conditions.^[20]^ Radiofrequency ablation is well established for dysplastic Barrett esophagus and early esophageal adenocarcinoma, demonstrating high rates of complete eradication of dysplasia.^[21,22]^

Cryotherapy and photodynamic therapy serve as alternative or adjunctive modalities, particularly in patients unsuitable for resection.^[23]^ These therapies are repeatable, tissue-sparing, and generally associated with low adverse event rates.^[24]^

### 3.4. Palliative interventions: stenting and biliary drainage

In advanced GI cancers, palliation of obstructive symptoms is essential to improve quality of life and permit ongoing systemic therapy.^[25]^ Self-expanding metal stents are widely used for malignant obstruction of the esophagus, gastric outlet, colon, and biliary tree, providing rapid symptom relief.^[26,27]^

Endoscopic retrograde cholangiopancreatography remains the standard for malignant biliary obstruction; however, EUS-guided biliary drainage has emerged as a safe and effective alternative when endoscopic retrograde cholangiopancreatography fails.^[28]^ These interventions reduce the need for percutaneous drainage and surgical bypass.

### 3.5. Advanced imaging and artificial intelligence

Image-enhanced endoscopy, including narrow band imaging, chromoendoscopy, confocal laser endomicroscopy, and volumetric laser endomicroscopy, improves detection and characterization of early neoplastic lesions.^[29,30]^

Artificial intelligence (AI) has rapidly gained prominence in endoscopy, improving lesion detection, optical diagnosis, and quality metrics.^[31]^ AI-assisted systems reduce miss rates, interobserver variability, and procedural inconsistency, with growing evidence supporting their integration into routine practice.^[32]^

### 3.6. Strengths, limitations, and future directions

The primary strength of this review lies in its clinically focused synthesis of recent, practice-changing advancements in interventional gastroenterology. However, limitations include its narrative design, potential selection bias, and lack of quantitative analysis.

Future progress will depend on standardized training, broader access to advanced endoscopic technologies, and high-quality clinical trials evaluating long-term outcomes and cost-effectiveness.^[33]^ Emerging innovations such as robotic endoscopy, drug-eluting stents, and endoscopic molecular diagnostics are likely to further expand the therapeutic scope of interventional GI oncology^[34,35]^ (Table 3).

**Table 3:** Emerging technologies in interventional GI oncology.

Technology	Description	Potential impact
AI-assisted endoscopy	Real-time polyp detection classification	Improves diagnostic accuracy, reduces miss rates
Liquid biopsy via endoscopic techniques	Sampling for circulating tumor DNA (ctDNA)	Noninvasive molecular profiling
Drug-eluting stent	Localized chemotherapy delivery	Combines palliation with targeted therapy
Robotic endoscopy	Precision-controlled endoscopic manipulation	Enhances complex resection and access

AI = artificial intelligence, GI = gastrointestinal.

## 4. Conclusion

Interventional gastroenterology has become an essential component of modern GI cancer management, offering minimally invasive diagnostic, curative, and palliative solutions across the disease spectrum. Techniques such as EMR, ESD, EUS-guided therapies, ablative modalities, and AI-enhanced imaging have improved patient outcomes while reducing morbidity. Despite challenges related to training and accessibility, continued innovation and multidisciplinary collaboration will further establish interventional gastroenterology as a cornerstone of comprehensive GI cancer care.

## Author contributions

**Software:** Gaurang Hasmukhbhai Suhagiya, Ali Karim, Sajid Ali, FNU Karishma.

**Conceptualization:** FNU Priyanka, Ali Karim, Alfa Almani, Allah Warayo, Hafsah Ali, Falah Asad, Kishore Kumar, Barkha Goswami, Sana Memon, Mahesh Kumar.

**Investigation:** FNU Priyanka, Zainab Obaid Ullah, Syed Ahmad Farooqi, Alfa Almani, Sajid Ali, Sana Memon.

**Validation:** FNU Priyanka, Zainab Obaid Ullah, Mubeena Javed.

**Project administration:** Zainab Obaid Ullah.

**Methodology:** Mubeena Javed.

**Formal analysis:** Allah Warayo.

**Data curation:** Barkha Goswami.

**Resources:** Abida Perveen.

**Visualization:** Abida Perveen.

**Writing – original draft:** Gaurang Hasmukhbhai Suhagiya, FNU Priyanka, Zainab Obaid Ullah, Syed Ahmad Farooqi, Ali Karim, Mubeena Javed, Alfa Almani, Sajid Ali, Allah Warayo, Hafsah Ali, FNU Karishma, Falah Asad, Kishore Kumar, Barkha Goswami, Sana Memon, Mahesh Kumar.

**Writing – review & editing:** Gaurang Hasmukhbhai Suhagiya, FNU Priyanka, Zainab Obaid Ullah, Syed Ahmad Farooqi, Ali Karim, Mubeena Javed, Alfa Almani, Sajid Ali, Allah Warayo, Hafsah Ali, FNU Karishma, Falah Asad, Kishore Kumar, Barkha Goswami, Sana Memon, Mahesh Kumar, Abida Perveen.
